# Structural Dynamic of a Self-Assembling Peptide d-EAK16 Made of Only D-Amino Acids

**DOI:** 10.1371/journal.pone.0002364

**Published:** 2008-05-28

**Authors:** Zhongli Luo, Xiaojun Zhao, Shuguang Zhang

**Affiliations:** West China Hospital, Laboratory for Nanobiomedical Technology, Sichuan University, Chengdu, Sichuan, China; Swiss Federal Institute of Technology Lausanne, Switzerland

## Abstract

We here report systematic study of structural dynamics of a 16-residue self-assembling peptide d-EAK16 made of only D-amino acids. We compare these results with its chiral counterpart L-form, l-EAK16. Circular dichroism was used to follow the structural dynamics under various temperature and pH conditions. At 25°C the d-EAK16 peptide displayed a typical beta-sheet spectrum. Upon increasing the temperature above 70°C, there was a spectrum shift as the 218 nm valley widens toward 210 nm. Above 80°C, the d-EAK16 peptide transformed into a typical alpha-helix CD spectrum without going through a detectable random-coil intermediate. When increasing the temperature from 4°C to 110°C then cooling back from 110°C to 4°C, there was a hysteresis: the secondary structure from beta-sheet to alpha-helix and then from alpha-helix to beta-sheet occurred. d-EAK16 formed an alpha-helical conformation at pH0.76 and pH12 but formed a beta-sheet at neutral pH. The effects of various pH conditions, ionic strength and denaturing agents were also noted. Since D-form peptides are resistant to natural enzyme degradation, such drastic structural changes may be exploited for fabricating molecular sensors to detect minute environmental changes. This provides insight into the behaviors of self-assembling peptides made of D-amino acids and points the way to designing new peptide materials for biomedical engineering and nanobiotechnology.

## Introduction

Chirality plays an absolutely central role in all life forms. Although there are chemically possible D- and L- amino acids, natural proteins are made of only L-amino acids. There is an intense and wide spread interest in understanding the origin and selection of chirality in prebiotic molecular evolution and biology. Why the chirality of life is asymmetric and why nature only uses L-amino acid and D-sugars are tantalizing questions.

In the past decades there has been much effort focused on the study of various self-assembling peptides and their relevance in biology, protein aggregation, and their applications in biotechnology and nanobiotechnology [1]–[5]. Not surprisingly most of this work has been carried out using the naturally occurring L-form amino acid [1]–[5].

It has been reported that some peptides undergo conformational change in the presence of external stimuli such as temperature, pH, ionic strength and metal ion binding manipulations [6]–[11]. Certain peptides were found to exhibited remarkable secondary structure plasticity and multifaceted behavior including the ability to undergo secondary structural transitions directly from beta-sheet to alpha-helix in response to changes in temperature or pH [reviewed in 12]. In other studies, peptides were shown to undergo structural transition between alpha-helix and beta-sheet through oxidation and reduction through incorporating several regularly spaced methionines of an 18-residue peptide [13]. In each of these cases the peptides studied were composed of naturally occurring L-form amino acids.

In 1992, a peptide motif with alternating hydrophilic and hydrophobic amino acids termed EAK16 (AEAEAKAKAEAEAKAK) was discovered from a yeast protein *Zuotin* that was originally found to preferentially bind to left-handed Z-DNA [14]. Initial computer modeling of this sequence showed the structure to be an alpha-helix: its lysines and glutamic acids on the side-chains with *i, i+3* and *i, i+4* arrangement could form potential ionic bonds. However another similar 16-residue peptide with the same amino acid composition with *i, i+3* and *i, i+4* arrangement, but with different sequence was again found to form an exceedingly stable alpha-helix [15], [16].

However, when the actual l-EAK16 peptide was studied following the reported method [15], [16] an unexpected result occurred. Instead of the expected alpha-helix the peptide formed an exceedingly stable beta-sheet structure. Further, it was found that this l-EAK16 peptide underwent spontaneous assembly to form well-ordered nanofibers and scaffolds. Thus, the first self-assembling peptides was discovered [1].

Subsequently numerous self-assembling peptides with various compositions, sequences, and length have been studied, largely all using peptides composed of L-amino acids. [1]–[7], [17], [18].

However, although there was a nagging question about its chiral counterpart, no serious attempt was made to study its mirror image form, namely, the self-assembling peptide made of only D-amino acids, until now.

We here report research that broadens understanding of this class of self-assembling peptides by studying a chiral mirror image peptide, d-EAK16 that is made of only D-amino acids of alternating hydrophilic and hydrophobic residues with sequence: Ac-A*E*A*E*A*K*A*K*A*E*A*E*A*K*A*K*-CO-NH_2_ (* denotes the D-amino acid). We report on its structural dynamics in terms of thermal stability, pH changes, ionic strength and chemically denaturing agents. Our findings suggest that the beta-sheet structure of the d-EAK16 has plasticity and is able to directly convert into an alpha-helical conformation under the stimulus of temperature or pH changes.

Peptides and proteins made of only D-amino acids are more stable since natural occurring proteases can degrade L-form peptide bonds, but cannot degrade D-form peptide bonds. Likewise, ribosomes can incorporate L-form amino acids but not D-form [19]–[23]. Nature has a remarkable ability for chiral selectivity [24]–[26]. It has been shown that a HIV protease made of all D-amino acids could only catalyze D-substrates [27].

Since mirror images of D-form peptide bonds resist natural enzyme degradation, D-peptide based materials will likely be more stable. Thus, a new class of D-form self-assembling peptides may prove to be very versatile in fabricating novel supramolecular architecture and have a wide range of applications in biotechnology, nanobiotechnology and medical technology.

## Results

### Molecular models of d-EAK16 and l-EAK16

**Figure 1:**
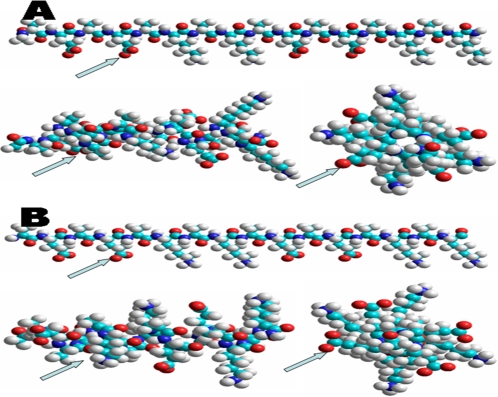
Molecular models of d-EAK16 and l-EAK16. They are a pair of chiral molecules with 3-D mirror image to each other. A) d-EAK16 and B) l-EAK16 are in the extended beta-strand (N–>C) (an alanine at N-terminus is on left and to a lysine at C-terminus on the right). The alpha-helical model, horizontal (left) and alpha-helical model looks from the center of axis (right) (alanine on N-terminus is on top and lysine on C-terminus on the bottom). Color code: hydrogen = white, carbon = cyan, oxygen = red and nitrogen = Blue.

We present two molecular models of the chiral EAK16 peptides (Fig. 1). The peptides have identical sequence but with different chiral form amino acids: D-amino acids, d-EAK16, (Fig. 1A) and L-amino acids, l-EAK16, (Fig. 1B). Although their structures look similar, their mirror image backbones and chemical properties proved to be very different.

### Structural characteristic of d-EAK16

**Figure 2:**
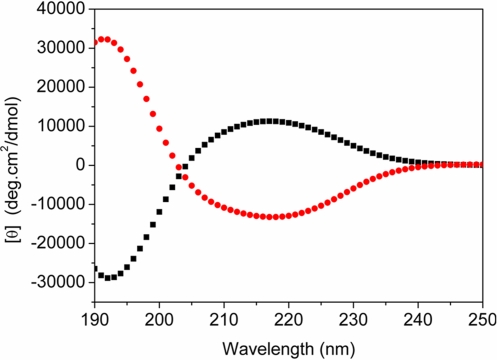
Circular dichroism spectra of peptides d-EAK16 and l-EAK16 were in water at 25°C. X-axis is wavelength in nm and Y-axis is expressed as mole residue ellipticity [θ]. The mirror images of d-EAK16 and l-EAK16 reflect the molecular chirality.

We have previously described a class of ionic self-assembling peptides made of only L-amino acids, which cannot only form stable beta-sheets but also undergo self-assembly to form macroscopic nanofibers scaffolds stained by Congo red [1], [3], [4]. We found in the current study that the D-amino acid chiral peptides similarly form stable beta-sheets with inverted CD spectra, a mirror image of the EAK16 peptide with L-amino acids (Fig. 2).

### Structural behaviors of chiral d-EAK16 peptide at different temperatures

**Figure 3:**
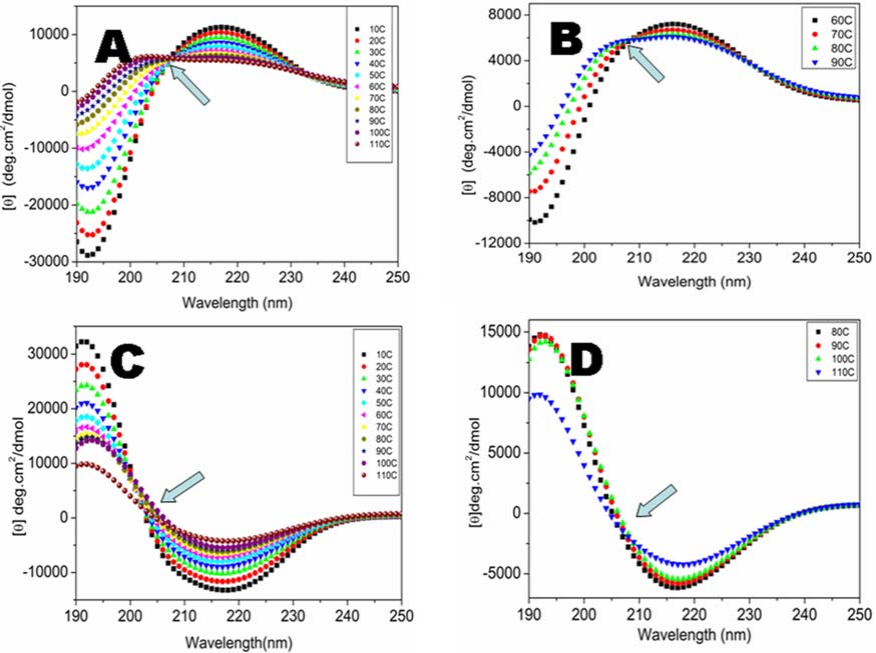
The temperature effect on the d-EAK16 and l-EAK16 (100 µM). Each spectrum was collected at indicated temperature from 10°C to 110°C, using the same samples covered with mineral oils to prevent water evaporation. Every temperature point was averaged from 3 measurements. The sample was incubated at indicated temperatures for 15 minutes because 3 at 5-minute each measurements took 15 minutes. After measurement at 10°C, then the temperature was increased to 20°C, 30°C, 40°C, etc in the CD instrument to reach the indicated temperature and eventually elevated to 110°C. A) The CD spectra of d-EAK16 from 10°C to 110°C. At 20°C, it formed a stable beta-sheet. This beta-sheet structure was stable until 80°C. It then underwent two distinctive transitions. During the first transition, the beta-sheet content was not only reduced, but also changed its twist. The second structural transition occurs between 80°C to 90°C and drastically between 90°C to 110°C. Here, the beta-sheet structure is converted to an alpha-helical structure. B) d-EAK16 exhibits 3 distinctive structures at different temperatures, 60°C, 80°C, and 90°C. The isosbestic point was at 208nm as indicated by an arrow. C) The CD spectra of l-EAK16 from 10°C to 110°C. It was a very stable beta-sheet. The beta-sheet content was reduced, but no obvious structural transition was observed. D) l-EAK16 was very stable at different temperatures, 80°C, 90°C, 100°C and 110°C. No drastic structural transition was observed.

A sample of 100 µM d-EAK16 was used for studying the peptide structures. Circular dichroism (CD) was used to follow the structural behaviors. The d-EAK16 peptide showed interesting structural dynamic at different temperature. The beta-sheet structure of d-EAK16 is relatively stable at low temperature up to 70°C. But, further increasing temperature resulted in an abrupt structural transition from beta-sheet to alpha-helix (Fig. 3). This observation is similar to what we have reported of other peptides [6], [7].

d-EAK16 first decreased its beta-sheet content at 60°C as observed from reduced ellipticity at 218 nm. This decrease may be resulted in a change in beta-backbone twist as shown by an increased ellipticity at 193 nm when the temperature increased beyond 60°C. At 70°C onset of a structural conversion was observed as the 218 nm valley widens toward 210 nm, and the ellipticity at 193 nm began to take on alpha-helical characteristics. On raising the temperature above at 80°C, an abrupt structure transition occurred in the CD spectra from a beta-sheet to an alpha-helix without a detectable random-coil intermediate (Fig. 3B). There was still a considerable amount of beta-sheet structure; yet, the widening of the valley toward 208 nm suggests increasing helical content. By 90°C, the structural conversion was nearly complete and a characteristic helical profile was observed similar to that previously reported for l-EAK12 and l-DAR16-IV* [6], [7]. No significant structural change occurred in at temperature change from 90°C to 110°C (Fig. 3A).

Such extreme thermal stability is unusual since most proteins, except for the thermophilic ones, can be denatured above 65°C. However, the d-EAK16's chiral counterpart l-EAK16 was very stable (Fig. 3C, 3D) [2]. The CD spectra signal showed only ∼20% decrease at 218 nm when the temperature was increased from 25°C to 90°C [2].

### Hysteresis

**Figure 4:**
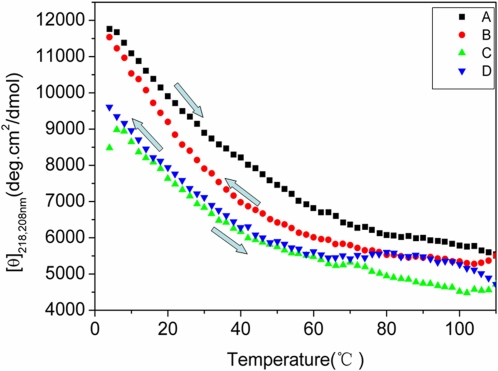
The thermal behavior of d-EAK16. A d-EAK16 peptide sample was measured at 218 nm and 208 nm from 4°C to 110°C with incremental 2°C change. The temperature equilibration time is 30 seconds and averaging time is 1 second. After reaching 110°C and waiting for 1-minute, the cooling from 110°C to 4°C was initiated. The changes of ellipticity from 4°C to 110°C and from the 110 to 4°C were shown. After the temperature cycles, [θ]_218nm_ of d-EAK16 decreased ∼2%, but increased ∼13% at [θ]_208nm_. A and B profiles refer to d-EAK16 ellipticity [θ]_218nm_ from 4°C to 110°C and back from 110°C to 4°C. On the other hand, C and D profiles refer to ellipticity [θ]_208nm_ from 4°C to 110°C and back from 110°C to 4°C.

In order to detect changes of the d-EAK16 at wavelength 218 nm and 208 nm, we carried out finer temperature step measurement, sampling at every 2° from 4°C to 110°C and from 110°C back to 4°C (Fig. 4).

We found an ∼48% decrease in signal at 218 nm when the temperature rose from 4°C to 80°C (base line 4°C) and an ∼9% decrease from 80°C to 110°C (base line 80°C, Fig. 4A). Similarly there was an ∼40% decrease in intensity at 208 nm when temperature rose from 4°C to 80°C and a 4.7% decrease from 80°C to 110°C (Fig. 4C).

However when the temperature was lowered from 110°C to 80°C (base line 110°C), there was an ∼4% increase in intensity at 218 nm and an ∼19% increase in intensity at 208 nm while an ∼70% increased was observed by raising the temperature from 80°C to 4°C (Fig. 4B).

When the temperature was raised from 4°C to 110°C and then reversed one minute later, (110°C to 4°C) the intensity decreased ∼2% at 218 nm and increased ∼13% at 208 nm (Fig. 4D). This observation suggests that the structural conversion is kinetically reversible.

### Long-term stability of d-EAK16 at high temperature

**Figure 5:**
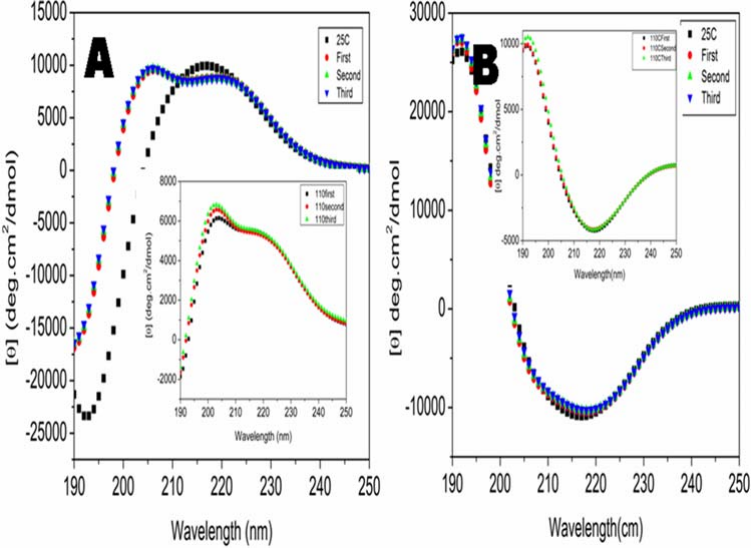
Temperatures induced d-EAK16 and l-EAK16 structural transition. d-EAK16 and l-EAK16 were incubated at 110°C and cooled to 25°C and the cycles were repeated 3 times. A) The d-EAK16 formed stable beta-sheets when measured at 25°C. The d-EAK16 underwent two-phase transitions when elevated at 110°C, from beta-sheet to alpha-helix. The figure insert is the time-dependence of d-EAK16 structural transition. The structural transition occurred in 3 stages. After first incubation, the beta-sheet became less stable and showed signs of structural change. After second incubation, it abruptly converted from a beta-sheet to an alpha-helix. After third and prolonged incubation, no significant change for alpha-helix was observed. B) The l-EAK16 stayed as a stable beta-sheet. No abrupt structural transition was observed for l-EAK16 even when temperature was elevated to 110°C. The insert is the time-dependence of l-EAK16 structural transition.

We asked if d-EAK16 is stable after repeated temperature cycles between 10°C to 110°C. Figure 5A shows d-EAK16 structural transition in three consecutive temperature cycles. The structural changes were recorded at an increase and decrease of temperature. Specifically, when d-EAK16 was incubated at 110°C, the first measurement was taken. It was then cooled to 25°C and the measurement was then repeated 2 times. The transition occurred at the first cycle when the stable beta-sheet became less stable and showed signs of structural change. After the second cycle, it abruptly converted from a beta-sheet to an alpha-helix. During the third incubation there was no additional alpha-helical change. Figure 5A shows d-EAK16 incubated at 110°C, cooled to 25°C, then heat to 110°C again, and measurement was taken at 110°C. It is interesting to point out that three repeated temperature cycles showed similar structures during incubation for different time periods.

Furthermore, beta-sheet to alpha-helical transition was observed after prolonged heating and the transition was completed after 3 cycles of temperature from 110°C to 25°C (Fig. 5A). However once the alpha-helical structure was formed, prolonged incubation did not further promote the transition; the structure remained very stable (Fig. 5A). On the other hand, the l-EAK16 is very stable, 3 repeated temperature cycles did not significantly change its beta-sheet structure (Fig. 5B).

**Figure 6:**
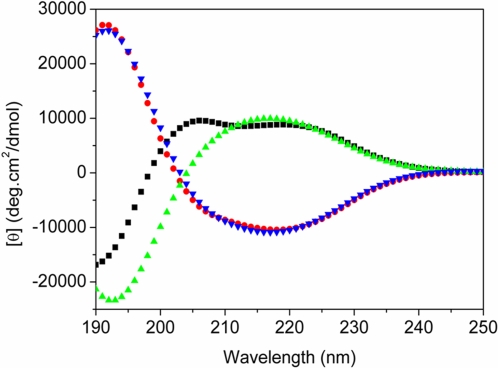
Comparison of structural behaviors of d-EAK16 and l-EAK16. Although both peptides have identical sequences, their chiral amino acids are mirror images. In l-EAK, there was no obvious structural change at 110°C and return to 25°C (red and blue). On the other hand, d-EAK16 exhibited drastic structural change at 110°C and back to 25°C. Initially at 25°C, it had a beta-sheet structure (green spectrum); however, after heating, it converted to typical alpha-helix structure and remained stable even after cooling to 25°C (black spectrum).

The temperature study of d-EAK16 and l-EAK16 is summarized in Figure 6. Although both peptides have identical sequences, their chiral amino acids are mirror images. For l-EAK, there was neither obvious structural change at 110°C, nor changes after return to 25°C (red and blue). On the other hand, d-EAK16 exhibited drastic structural change at 110°C. Initially at 25°C the peptide had a beta-sheet structure (green spectrum); however, after heating, it converted to typical alpha-helix structure (black spectrum) and remained stable even after cooling to 25°C. This phenomenon is very similar to our previous finding of two peptides made of only L-amino acid [6], [7].

### Effect of pH

**Figure 7:**
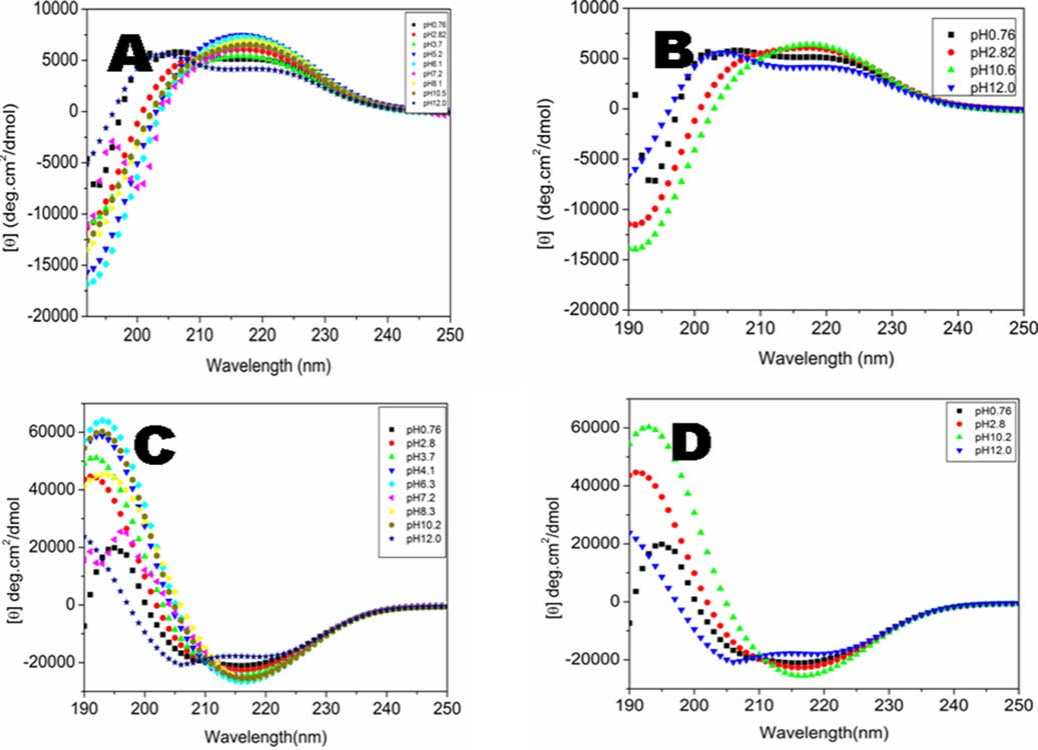
pH effect on the d-EAK16 and l-EAK16 structural transition. The d-EAK16 was incubated in solutions of various pH range from 0.76–12.0. A) The structural behavior from the d-EAK16 spectra in different pH showed the variations. The structures were different at pH0.76, pH12.0 and neutral pH. The d-EAK16 beta-sheet content is fairly similar at pH 5.2, pH6.1, pH7.2, pH8.1 and pH10.5. The d-EAK16 at pH2.8 and pH10.5 exhibits somewhat different spectra. There seemed to be either an intermediate structure or mixture with both alpha-helix and beta-sheet. B) The d-EAK16 spectra suggest four variations of structures at pH0.76, pH2.8, and pH10.5 and pH12.0. C) The structural behavior from l-EAK16 spectra in different pH showed variations. D) The l-EAK16 spectra suggest four variations of structures at pH0.76, pH2.8, pH10.2 and pH12.0.

It is well known that pH changes have drastic effects on protein and peptide structures [28]–[30]. Much of such work was systematically carried out using polymeric peptides [29]. We show that d-EAK16 also underwent conformational changes as a function of pH changes [Fig. 7].

The side chains of glutamic acids and lysines on d-EAK16 have ∼p*K*a4 and ∼p*K*a10, respectively. We first tested two extreme pH conditions, pH0.76, which is below the p*K*a of glutamic acid, and pH12, which is above the p*K*a of lysine, under such condition glutamic acid is protonated and lysine is deprotonated, respectively. From pH3.7 to pH7.0 d-EAK16 exhibited mirror image of the conventional L-amino acid type beta-sheet spectrum: a minimum ellipticity at 218 nm and a maximum at 190 nm with a noticeable change in the peptide CD profile. The d-EAK16 formed into beta-sheet when the pH value was close to its pI value. It is worth noting that when d-EAK16 was fully protonated below pH1, its structure formed an alpha-helix; when at pH2.8, there was a tendency toward an alpha-helix. This suggests that there might be either some structural intermediates or both beta-sheet and alpha-helical structures might coexist, which was different from the study of poly-L-lysine [29].

Figure 7 shows the pH effect on the d-EAK16 structure dynamics. The d-EAK16 was incubated in solutions ranging from pH0.76–12.0. The spectra of d-EAK16 showed its structural variations in different pH. The beta-sheet content was different at pH2.8 and pH7.2. The beta-sheet contents were similar at pH3.7, pH5.2, pH6.1, pH7.2 and pH8.1. There existed a similar backbone twist at acidic pH0.76 and basic pH12.0 as shown in the 190 nm region. There seemed to be either an intermediate or a mixture structure with both helical and sheet character, however, it is difficult to distinguish the two at present time and more systematic studies are required to clarify this. The CD spectra and AFM images suggest several different structures of d-EAK16 at pH0.76, pH2.8, pH10.6 and pH12.0 (Fig. 7, and further below).

At both acidic pH0.76 and basic pH12.0 d-EAK16 formed alpha-helical structures (Fig. 7A). When the pH was increased from pH10.6 to pH12 (Fig. 7B), the lysines on d-EAK16 became de-protonated and the structure became more helical. At pH10.6 a beta-sheet spectrum of d-EAK16 was clearly observed. At pH12.0 there was a noticeable change in the peptide CD spectrum, suggesting an altered backbone conformation. Thus, d-EAK16 followed the same trend as the well-characterized poly-L-lysine, in which higher pH values increase the tendency to form an alpha-helix (Fig. 7B).

### Effect of ionic strength and denaturing agents

**Figure 8:**
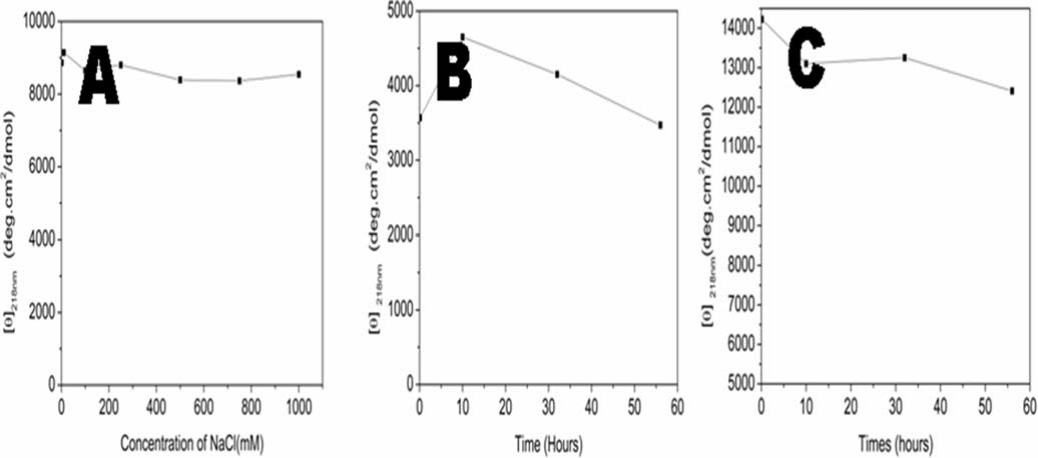
Influence of the ionic strength and denaturing agents. A) The effect of NaCl. The sample solution was incubated at various concentration of NaCl for ∼2 hours. B) d-EAK16 was stable in 8 M urea, which is similar as previously report for l-EAK16. C) Effect of 0.1% SDS, likewise, it had little denaturing effect, similar as previously report of l-EAK16.

We studied the salt influence on the d-EAK16. There was no drastic ellipticity change at 218 nm for d-EAK16 in a wide range of NaCl concentration from 1, 10, 100, 250, 500, 750 to 1000 mM (Fig. 8A). It is known that NaCl promotes peptides to form beta-sheets by reducing electrostatic repulsion [1], [2], [31]. Both d-EAK16 and the l-EAK16 were stable in NaCl solution.

The effect of denaturing agents 8 M urea, 6 M guanidine.HCl, and 0.1% SDS were examined. The beta-sheets structure of d-EAK16 was not significantly destabilized in 8 M urea, which is consistent with previous observation of l-EAK16 [2]. Figure 8B shows the plots of [θ]_218nm_ from the CD measurement of a few minutes to 10, 32 and 56 hours. We found the residue ellipticity at [θ]_218nm_ was only slightly changed with prolong time. Likewise, d-EAK was not notably denatured in 0.1% SDS (Figure 8C) and there was very little change of the residue ellipticity at [θ]_218nm_, similar as observed for l-EAK16 [2].

### Study d-EAK16 material structure using AFM and optical microscopy

**Figure 9:**
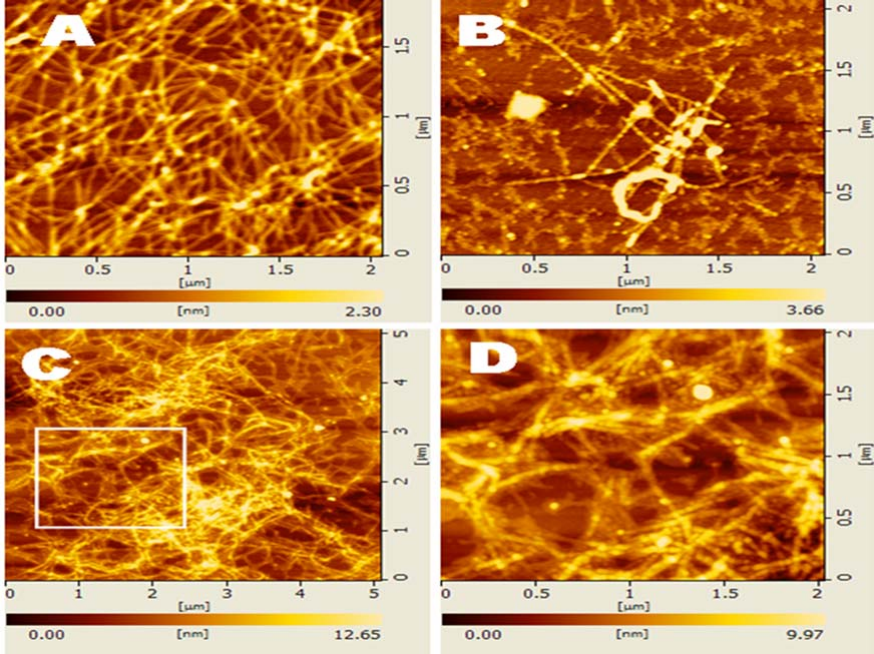
The AFM images of d-EAK16 (1 mg/ml, 0.1%) under repeated cycles of thermal treatment. A) d-EAK16 solution was incubated at 25°C for 4 hours and then PBS was added to self-assembling overnight, B) d-EAK16 solution was incubated at 100°C for 4 hours and PBS was added to self-assembling overnight, C) d-EAK16 solution was incubated at 100°C for 4 hours and PBS was added to allow self-assembly for 2 nights, D) higher magnification of the image inside the white box C).

As previously reported, l-EAK16 forms well-ordered nanofibers with well-defined structure [1]. We asked if the chiral form d-EAK16 could also form well-ordered nanofibers. AFM observations revealed that d-EAK16 does indeed form ordered nanofibers ranging in length from a few hundred nanometers to a few microns when the peptide was allowed to self-assemble in PBS overnight (Fig. 9).

**Figure 10:**
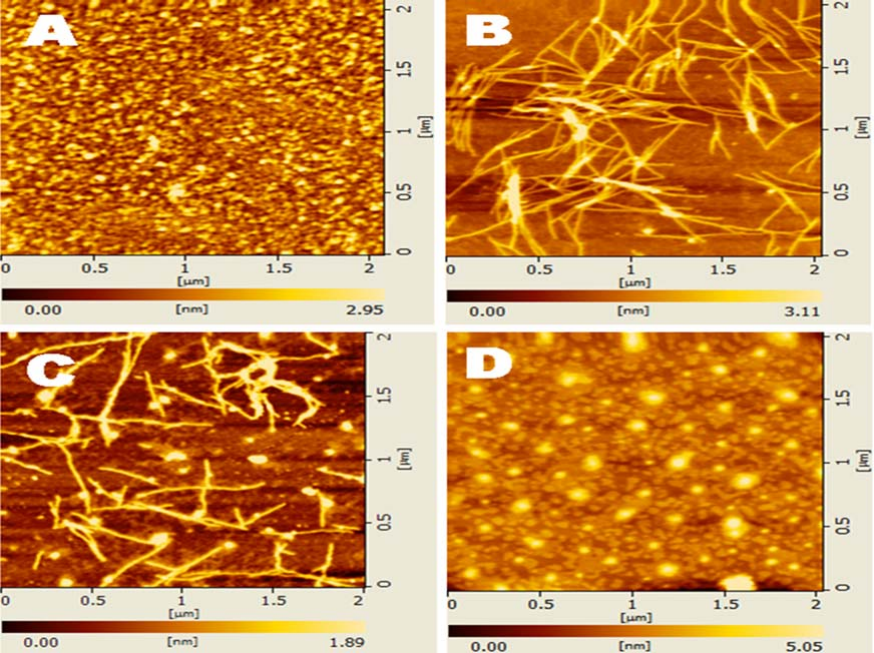
pH sensitivity. The AFM images of d-EAK16 (100 µM) are displayed under different pH conditions. A) pH1, B) pH7, C) pH10.6, D) pH12.8. When at pH1 or pH12.8, the peptides aggregated to form the globular structure, when at pH10.6 globular and nanofibers were formed; but at pH7, nanofibers were observed.

**Figure 11:**
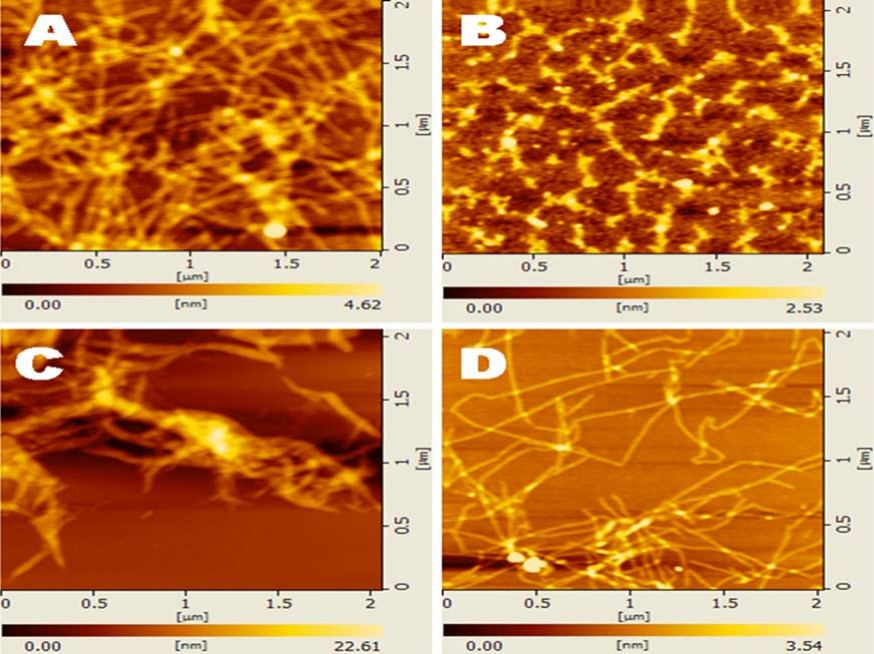
The AFM images of d-EAK16 (1 mg/ml, 0.1%) under salt and denaturation agents. A) 1 M NaCl, B) 1% SDS,C) 8.1 M urea, D) 7.1 M guanidine.HCl. d-EAK16 was incubated in the denaturation agents or salt overnight.

AFM examinations revealed these nanofibers further form 3-D scaffolds in the presence of salts. The concentration of ionic strength was important for d-EAK16 to form nanofibers. These observations suggested the peptide chirality alone did not hinder self-assembly nor nanofiber formations of d-EAK16 (Fig. 9, Fig. 10 and Fig. 11).

We observed that the peptides made of D-amino acids with beta-sheet structure could self-assemble into ordered nanofibers (Fig. 9A, Fig. 10B and Fig. 11A). The nanofibers were sensitive to pH conditions. At acidic pH1.1 d-EAK16 formed globules (Fig. 10A); at neutral pH7.2, it formed nanofibers (Fig. 10B); at basic pH10.6, it formed mixed aggregates and nanofibers (Fig. 10C); at pH12.0, it formed only aggregates (Fig. 10D). The results suggest the fine structures of the d-EAK16 materials were responsive in different pH environment.

**Figure 12:**
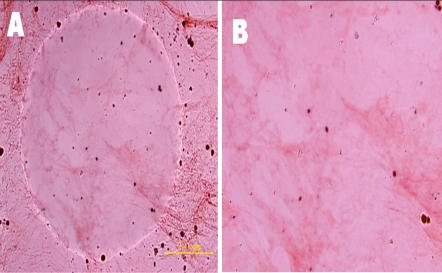
Optical microscopy images of d-EAK16 membranes. The colorless membranous structure was formed in phosphate-buffered saline and transferred to a glass slide. It can be seen by the naked eye when stained with Congo red. A) At 100 magnifications, the circular region is the membrane in the water and the rest is the dry membrane in the air at 20°C, B) at 200 magnifications, the membranes in water of A).

In the presence of monovalent salts, d-EAK16 was also able to spontaneously self-assemble to the transparent macroscopic material that can be stained with Congo red (Fig. 12 A and B), similar as observed for l- EAK16 [1], [3].

Our observations suggest that the peptide chirality alone is not crucial for nanofiber formation. This is not surprising since both peptides are made of the same amino acid isomers where every residue in the peptide is either a D- or L- isomer. It suggests that chirality for the spatial arrangements is allowed and the weak noncovalent interactions are more important. Chirality alone does not interfere with noncovalent interactions nor influence the beta-sheet structure and the peptides self-assembly to form nanofibers.

However the peptides made of hetero-chiral amino acids, namely, a sequence with alternating L-amino acids and D-amino acids could not undergo self-assembly to form the nanofibers. This is probably due to their incompatible chiral and structural features, largely affected by the chirality. Detailed studies will be reported elsewhere.

## Discussion

Our studies show that the chirality of peptides does not interfere with the molecular self-assembly nor interferes the formation of well-defined nano-structures. We believe that molecular self-assembly is a fine-tuned balance between numerous noncovalent weak interactions. These include: 1) hydrogen bonds, 2) electrostatic interactions, 3) hydrophobic interactions, 4) van der Waals force and 5) water-mediated-hydrogen bonds. Each interaction in isolation is rather weak, but collectively they exert strong molecular forces to facilitate formation of molecular structures and to maintain the stability of these structures.

### Effect of chiral peptide sequence

Our study suggests that amino acid sequence and ionic complementarity contribute to the overall propensity to form a specific secondary structural behavior and plasticity, as well as to the stability of the secondary structure.

The helical dipole moment likely plays an important role for the structural plasticity. The sequence Ac-AEAEAKAKAEAEAKAK-CONH_2_ with 2 negatively charged glutamic acids (E) close to the N-terminus and 2 positively charged lysine (K) at the C-terminus might balance the helical dipole moment thus may have a tendency to stabilize helical structures [32].

### Temperature effect

In our observations of the effect of temperature, pH, ionic concentration, and the denaturation agents, only the thermal behavior of d-EAK16 is different from its L-peptide counterpart. For d-EAK16 there is a structural hysteresis. When the temperature cycled from 4°C to 110°C its beta-sheet converted to an alpha-helix and from 110°C to 4°C alpha-helix could again reverted back to the beta-sheet form. This suggests that d-EAK16's structural change was as a function of temperature. When temperature was at 10°C, CD spectra of d-EAK16 and l-EAK16 and l-EAK16 were almost perfect mirror images (Fig. 2). But at 110°C, they were not symmetrical (Fig. 3, Fig. 5, Fig. 6). This observation suggests the subtle structural plasticity of these chiral peptides with the identical sequences.

The secondary protein structural units are generally believed to be stable such as alpha-helices and beta-sheets [33]–[36]. However, our study suggests these units are structurally dynamic. This observation is consistent with the notion that when proteins interact with their ligands, other molecules and proteins, they need to change their conformation to adopt the new structure so as to accommodate the entities, similar as place gloves on hands.

We showed that d-EAK16 at elevated temperature underwent a structural transition from a beta-sheet directly to an alpha-helix without an observable random coil intermediate (Fig. 3A and Fig. 5A). Thus the secondary structure of the self-assembling peptide had drastically changed as a function of temperature. At elevated temperature, the beta-sheet spectrum was replaced by an alpha-helix spectrum. We wonder if such an event could also take place *in vivo* during protein folding, protein-protein interaction, and protein transportation, especially for those thermophilic bacteria and other microbial that live in elevated temperature environment.

In our study it appeared that d-EAK was not as stable as l-EAK (Fig. 3, Fig. 5 and Fig. 6). When d-EAK and l-EAK were cooled from 110°C to 25°C, we observed that the l-EAK did not have appreciable change (Fig. 5B). On the other hand, the secondary structure of the d-EAK was significantly changed (Fig. 5A, Fig. 6). It is plausible that the D-amino acids may not be as stable as L-amino acid so that the D-amino acids might affect a subtle balance between noncovalent interactions. Additionally, our results show that l-EAK is very stable at 90°C (Fig. 3B). It is very stable even at 110°C for 2 hours (Fig. 5B and insert, Fig. 6) and its beta-sheet spectrum was not significantly changed.

When d-EAK16 solution was at room temperature (Fig. 9A), no unusual situation was observed. However, when it was incubated at 100°C for 4 hours, some peptide structural transition from beta-sheet directly to an alpha-helix occurred. However, when PBS (10 mM) was added to the peptide solution overnight, some nanofibers were obtained (Fig. 9B). After continuous incubation for 2 days, almost all peptides formed nanofibers (Fig. 9C, Fig. 9D). The results suggest the secondary structure of d-EAK16 is stable, and its quaternary structure is little affected by temperature.

Interestingly, a recent primitive earth model of the silicon cycle in the precambrian era suggests that in some areas seawater temperature could be as high as ∼70°C [37] due to Earth temperature instability. It is tempting to suggest that the L-amino acids and L-peptides were plausibly just slightly more stable than those of D-peptides during the prebiotic molecular evolution in such environment.

### pH effect

Our previous observations have shown that when l-EAK16 is incubated in pH1.5, pH3.0, pH7.0, and pH11 the beta-sheet is not significantly affected [2]. Our current study shows a similar result in its chiral mirror: d-EAK16 incubated in pH3.7, pH5.2, pH6.1, pH7.2, pH8.1 for ∼2 hours also formed beta-sheet. Both d-EAK and l-EAK16 are amphiphilic and contain 4 negatively charged glutamic acid and 4 positively charged lysine residues and may be able to buffer H^+^ or OH^−^ in solutions. When at pH0.76, the l-EAK16 formed a beta-sheet, but the d-EAK16 formed an alpha-helix. At pH12.0 both l-EAK16 and d-EAK16 beta-sheet became less stable and showed signs of structural change. It seems that the d-EAK16 is more sensitive to pH changes than that of its chiral l-EAK16 (Fig. 7).

### Other studies

Previously other investigators have reported on studies of water soluble synthetic D-peptide hormones, including oxytocin [24] bradykinin [38] and angiotensin [39]. These peptides do not undergo self-assembly. Furthermore, only a few examples of synthetic enantiomers of naturally occurring peptide antibiotics are known, such as, enantio-enniatin B [40]–[43]. It has not been reported that they can undergo self-assembly in solutions.

Our findings demonstrate that the d-EAK16 can indeed undergo self-assembly to form nanofibers and membrane at 20°C. A transparent membrane was seen when viewed under a 20X phase-contrast microscope. The membrane can be stained by Congo red dye that preferentially stains beta-sheet. The membrane was visible in phosphate-buffered saline (Fig. 12). The self-assembling peptide systems occur with both chiral amino acids. Thus, it is possible to design self-assembling peptides using L- and D-amino acids [1].

### Other implications

The peptide d-EAK16 is more temperature sensitive than its chiral counterpart. The transition from beta- to alpha- and alpha- to beta- act like a molecular switch with dramatic conformational change. A slight conformational change in secondary structural units, alpha-helices, and beta-sheets, could have significant consequences in determining the overall protein structure and therefore its biological function. The structural dynamics may result in a considerable geometrical change might occur in proteins with significant shape changes. It seems that both d-EAK16 and l-EAK16 are sensitive to external stimuli such as pH, temperature and ionic concentration for their conformational changes.

Since large numbers of beta-sheets have been found in a wide spectrum of amyloid plaque formation, our studies on the structural transition from an alpha-helix to a beta-sheet may be relevant to understanding molecular behaviors of some amyloid peptides.

d-EAK16 can undergo self-assembly to form nanofiber scaffolds that could be useful as a class of designer extracellular matrices (ECM) for 3-D culturing tissue cells. They are well suited as hydrogel scaffolds that can be fabricated and shaped to fill anatomical defects for use in tissue repair. Their architecture may be designed to provide the desired properties necessary to support a cell for a number of studies in a 3-D environment (Fig. 9).

In addition, once the nanofibers of the d-EAK are formed (Fig. 9B, Fig. 10A and Fig. 11A), they are stable and resistant to digestion by several proteases including trypsin, a-chymotrypsin, papain and protease K (Z. Luo, unpublished results), making these peptides potentially useful for controlled drug delivery, as therapeutics and molecular medicines.

Some preliminary results suggest that the d-EAK16 may inhibit some bacteria, including *Escheriahia coli, Streptococcus pneummuce* and *Klebsiella pneummuce* (Luo, manuscript in preparation). Furthermore, the d-EAK form of nanofiber scaffolds can instantly stop organ bleeding in liver, skin, kidney and lung (Luo, manuscript in preparation).

In nature, molecular processes, including enzyme catalysis, cellular signaling, cell-cell communication, molecular replication and regulation, depend on a delicate, intricate and fine-tuned balance between molecular and supramolecular chirality [44].

Furthermore, during origin of life formation there perhaps existed two kinds of amino acids: one the L-form, and the other is D-form. There are now almost exclusively L amino acids in all living systems. Why should nature favorably chooses L- amino acid over their chiral D-form? Discovering the answer to this enigmatic question is now the subject of much active scientific pursuit [45].

## Materials and Methods

### Peptides synthesis and purification

Peptide d-EAK16 sequence is AcHN-A*E*A*E*A*K*A*K*A*E*A*E*A*K*A*K*-CONH_2_ (* denotes to D amino acid), and l-EAK16 sequence is: AcHN-AEAEAKAKAEAEAKAK-CONH_2_. These peptides were commercially custom-synthesized by solid-phase peptide synthesis (Chengdu CP Biochem Co., Ltd, Chengdu, China). The peptides were acetylated and amidated on the N-terminus and C-terminus, respectively. The peptides were purified by HPLC and characterized by mass spectroscopy. The purity of the d-EAK16 was 98.11% and of the l-EAK16, 95.02%. The lyophilized white powder was stored at 4°C. Stock solutions of the peptides were prepared at concentrations of 1.0 mg/ml in water (Millipore Milli-Q system) and stored at 4°C before to use.

### Circular Dichroism Spectroscopy

The samples consisted of 1 mg/ml peptide aqueous stock dissolved and adjusted to 100 µM in solution in water, pH buffer, or NaCl solution. A sample of 500 µl was used in a CD curvette with a 2 mm path-length. Measurements were carried out on an Aviv 400 CD spectrometer (Aviv Biomedical, Lakewood, NJ, USA). The samples were incubated at 10°C–110°C covered with 100 µl the mineral oils to prevent the solution evaporation.

The aqueous samples were measured from 190 nm to 260 nm at different temperature as indicated. The measurement was averaged over 3 seconds through the entire wavelength range.

To measure the effect of pH on alpha-helix and beta-stands wavelength spectra were measured for samples of d-EAK16 equilibrated 2-hours in phosphate buffer (pH with HCl or NaOH) at pH = 0.76, 2.8, 3.7, 5.2, 6.1, 7.2, 8.1, 10.6 and 12.0. CD spectra were converted to mean residue ellipticity.

The thermal behaviors of the peptide samples were examined through increasing or decreasing temperature and through equilibrating the samples from 4°C to 110°C and from 110°C back to 4°C with an increment of 2°C and 30-second temperature equilibrium.

The effect of ion concentration in NaCl solution was measured ranging from 1, 10, 100, 250, 500, 750 to 1000 mM. Denaturing agent experiment was carried out using 8 M urea ,0.1% SDS and 7.1 M guanidine.HCl.

### AFM

Aliquots of 5–10 µl were removed from the peptide solution at various times after reparation and deposited onto a freshly cleaved mica surface. The peptide solution was 0.1–10 mg/ml. To optimize the amount of peptide adsorbed, each aliquot was left on mica for 30–60 seconds and then washed with 100 µl at least three times using deionized water. The mica surface with the adsorbed peptide was then dried in air and imaged immediately. The images shown were obtained by scanning the mica surface in air by AFM (SPM400, Japan) operating in Tapping Mode. When imaging soft biopolymers with AFM at high resolution, it is important to minimize the tip tapping force. Soft silicon cantilevers were chosen (SI-DF2000, K-A102001604, Japan) with spring constant of 1–5 N/m and tip radius of curvature of 5 nm–10 nm. AFM scans were taken at 512×512 pixels resolution and produced topographic images of the samples, in which the brightness of features increases as a function of height. Typical scanning parameters were as follows: Scanner X/Y/Z: 53.00/53.00 /4.19 nm/N, Lever kz/kt/f0: 20.00 N/m/ 100.00 N/m/ 150.00 kHz, Length/Tip 200.0 µm/10 µm, Scan Mode: 2ch Simul, Data type: Topography (Servo), Area /Speed 1000×1000 nm/1.0–1.5 Hz, Amp. Ref.: −0.1 to −0.3, Vib.Voltage: 1.0–2.0 V, Bias: 0.000 V, Integral and Proportional gains 0.03–0.3 and 0.1–0.5 respectively.

### Peptide membrane preparations

The peptide membranes were prepared as follows: 5–10 µl of the stock solution of d-EAK16 peptide (10 mg/ml) was added to 0.5–1.0 ml of phosphate-buffered saline (150 mM NaCl/10 mM sodium phosphate, pH 7.4) with 0.00001% Congo red in a glass slide.

### Molecular modeling

Molecular models of these peptides were constructed using free modeling software from China (Hyperchem professional version 7.5, http://www.hyper.com). The software package was run on a PC machine.
